# First Report of Autochthonous Canine Leishmaniasis in Hong Kong

**DOI:** 10.3390/microorganisms10091873

**Published:** 2022-09-19

**Authors:** Jeanine Sandy, Anthony Matthews, Yaarit Nachum-Biala, Gad Baneth

**Affiliations:** 1 Department of Veterinary Clinical Sciences, City University of Hong Kong, Kowloon, Hong Kong; 2 Acorn Veterinary Hospital, G/F 68-80 Second St., Sai Yung Pun, Hong Kong; 3 Koret School of Veterinary Medicine, The Hebrew University of Jerusalem, Rehovot 91905, Israel

**Keywords:** canine, Hong Kong, *Leishmania infantum*, phlebotomine

## Abstract

Canine leishmaniasis is a zoonotic disease caused by *Leishmania infantum*; transmitted by the bite of phlebotomine sand flies. *Leishmania infantum* amastigotes were identified by cytology from a locally born Hong Kong dog exhibiting nasal, cutaneous, and systemic disease who was part of a kennel of eight dogs. All eight kennel dogs were subsequently tested serologically by enzyme-linked immunosorbent assay (ELISA) and by polymerase chain reaction (PCR) followed by DNA sequencing for *L. infantum* infection. The local dog was seropositive and blood and splenic tissue were PCR positive for *L. infantum* whilst the other kennel dogs were negative on serology and PCR. Autochthonous transmission was suspected for the local dog as Hong Kong lacks known vectors of *L. infantum*. Either vertical transmission from the deceased dam who had previously died with disease suspicious for leishmaniasis or horizontal transmission from a second non-locally born kennel dog who had been diagnosed previously with leishmaniasis was possible. This is the first recorded autochthonous case of canine leishmaniasis in Hong Kong. Leishmaniasis should be considered as a differential for cutaneous or systemic illness in local untraveled dogs in Hong Kong. In addition, as dogs serve as *L. infantum* reservoirs for human infection attention should be paid to the possibility of leishmaniasis emerging in Hong Kong.

## 1. Introduction

Leishmaniasis is an important disease of animals and humans which affects four eco-epidemiological regions of the world, including South-East Asia [1]. Canine and human leishmaniasis caused by *Leishmania infantum* are transmitted by the bite of phlebotomine sand flies. Infected dogs are the main domestic reservoir of the parasite for transmission to both humans and animals. Leishmaniasis is endemic in various parts of China [2] and transmitted in China by four different sand fly species: *Phlebotomus chinensis*, *P. longiductus*, *P. wui*, and *P. alexandri* [1]. According to the Hong Kong Food and Environmental Hygiene Department, the government body responsible for monitoring vector-borne diseases of humans, no human leishmaniasis cases have been reported in Hong Kong. There are currently no specific sand fly monitoring procedures by the department in Hong Kong but insect monitoring has identified various flies belonging to the Psychodidae with no *Phlebotomus* spp. found (personal communication; M. W. Lee, Pest Control Advisory Section, Food and Environmental Hygiene Department, Hong Kong). Canine leishmaniasis was diagnosed unexpectedly in a locally born dog, who had never left Hong Kong, residing in a kennel of eight dogs which included another imported dog previously found to have leishmaniasis but was clinically cured following treatment. Autochthonous transmission was suspected to have occurred either by horizontal or vertical mechanisms. 

## 2. Materials and Methods

**Figure 1:**
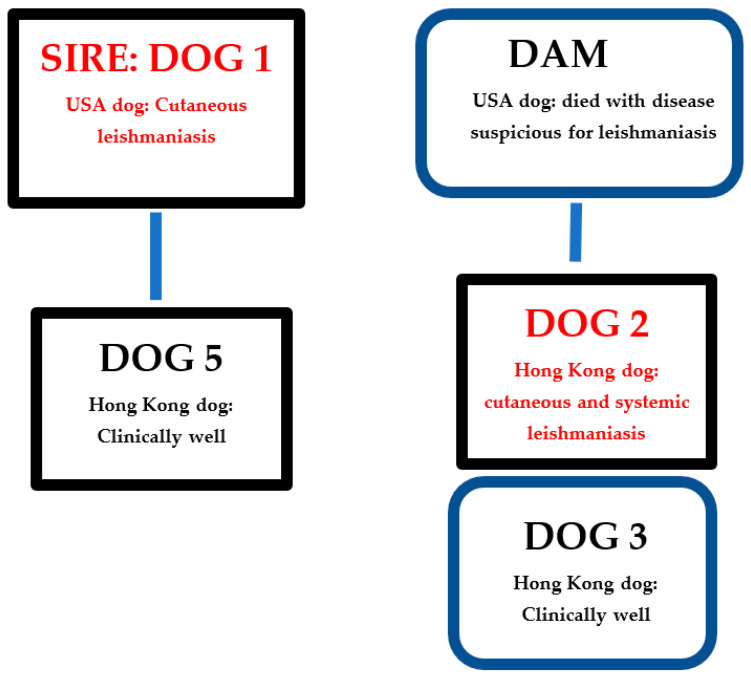
Leishmaniasis disease status and relationships of dogs within the Hong Kong kennel. USA: United States of America. Black rectangles: Male dogs. Blue rounded rectangles: Female dogs.

Eight privately owned, adult Belgian Malinois, 4 males and 4 females, aged between 6 and 10 years, resided on a single, Hong Kong Island property, working as guard dogs (Table 1). Four dogs were born in Hong Kong (dogs 2, 3, 5, 6), two in the Netherlands (dogs 4, 8), one in the United States of America (USA) (dog 1), and one in Sweden (dog 7). Two dogs (dog 2 and 3) were littermates, both born in Hong Kong to a dam imported from the USA and dog 5 was the progeny of dog 1 (Figure 1). All kennel dogs were under the veterinary care of author 2 (AM) and all testing and medical record retrievals were performed as part of disease investigation with permission from the owner. 

Over a 5-year period, four kennel dogs (dogs 1, 2, 3, 4) had presented to the attending veterinarian with dermatitis (dogs 1, 3, 4) or dermatitis with systemic illness (dog 2). Skin biopsies from dog 1 were collected for histopathology in 2015 along with an EDTA blood sample which was submitted for quantitative PCR to IDEXX Reference Laboratories, UK. In 2019, aspirates of spleen and liver were collected from the systemically ill dog (dog 2), which were stained with Wright’s Giemsa (WG). Following cytological identification of amastigotes in dog 2, additional aspirates of the spleen were placed into a sterile tube for PCR. Skin biopsies were collected in 2019, from 2 other kennel dogs with dermatitis (dogs 3, 4), with each sample split, with half of the sample placed into sterile saline for PCR, and the other half processed for histopathology. All biopsy samples for histopathology were fixed in formalin and processed into paraffin blocks and stained with haematoxylin and eosin (H and E), Grocott’s methenamine silver (GMS), periodic acid Schiff (PAS), Ziehl Neelsen (ZN), Gram and Giemsa for histology. Whole blood was collected from the cephalic vein of all eight kennel dogs between 2019 and 2020 for PCR and serology. PCR was also conducted on the paraffin block containing skin collected in 2015, with all tests performed by the Koret School of Veterinary Medicine, Hebrew University, Israel.

In summary, PCR for *Leishmania* detection was performed on EDTA blood from all eight dogs, punch biopsies of fresh skin placed into sterile saline from the two dogs with active skin disease (dogs 3, 4), paraffin-embedded skin from dog number 1 collected in 2015 when ulcerative skin disease first appeared, and aspirated splenic tissue collected in a plain tube from the systemically ill dog (dog 2). DNA was extracted from all blood and tissue samples using the Qiagen EZ1 Advanced XL automated system (QIAGEN GmbH, QIAGEN Strasse 1, 40724 Hilden, Germany). DNA was stored at −7 °C before being sent to the Hebrew University in Israel. DNA from the paraffin-embedded tissue was extracted from 3 different pieces of the same block using the Quick-DNA^TM^ FFPE kit (Zymo research, Irvine, CA, USA) according to the manufacturer’s instructions. 

The presence of *Leishmania* DNA in samples was tested using two PCR protocols for amplification of different targets. A 265 bp fragment of the *Leishmania* internal transcribed spacer 1 (ITS1) region of the *L. infantum* rRNA operon was amplified by real-time PCR using primers ITS-219 F (AGCTGGATCATTTTCCGATG) and ITS-219R (ATCGCGACACGTTATGTGAG) and then evaluated by high-resolution melt (HRM) analysis as previously described [3]. In addition, 120 bp of the *Leishmania* kinetoplast DNA (kDNA) minicircle were amplified by real-time PCR using primers JW11 (CCTATTTTACACCAACCCCCAGT) and JW12 (GGGTAGGGGCGTTCTGCGAAA) and then evaluated by melt curve analysis as previously described [3,4]. All positive PCR products were sequenced using the BigDye Terminator v3.1 Cycle Sequencing Kit and an ABI PRISM 3100 Genetic Analyzer (Applied Biosystems, Foster City, CA, USA), at the Center for Genomic Technologies, Hebrew University of Jerusalem, Israel. DNA sequences were evaluated with the ChromasPro software version 2. 1.1 (Technelysium Pty Ltd., Brisbane, QLD, Australia) and compared for similarity with sequences available in GenBank^®^, using the BLAST program (http://www.ncbi.nlm.nih.gov/BLAST/, accessed on 10 September 2022).

*Leishmania* serology was performed on blood samples collected from the cephalic vein of all 8 dogs collected between 2019 and 2020. Serum was stored at −70 °C before being sent to the Koret School of Veterinary Medicine, Hebrew University, Israel. Serology for anti-leishmanial antibodies was performed by enzyme-linked immunosorbent assay (ELISA) using *L. infantum* antigen, as described previously [5]. Dog 2 had a previous round of serology with the first blood sample collected at the time of systemic disease emergence, which was sent to the Veterinary Medical Diagnostic Laboratory at Texas A&M University, College Station, TX, USA.

## 3. Results

**Figure 2:**
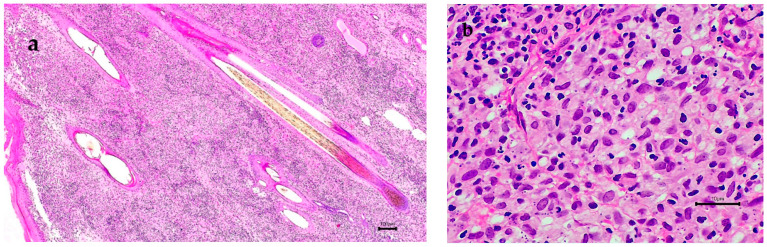

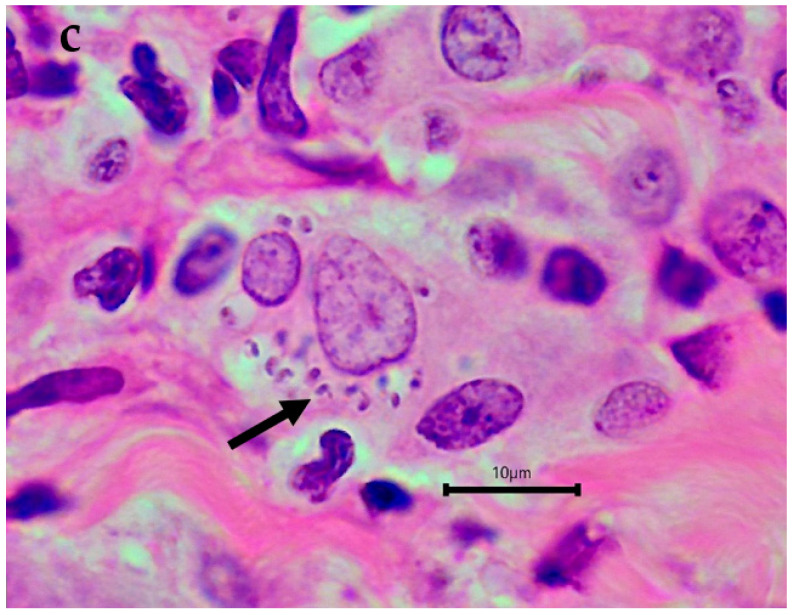
Nail bed skin histopathology from dog 1 collected in 2015: (**a**) Granulomatous dermatitis. Haematoxylin and eosin staining; bar = 100 um. (**b**) Granulomatous dermatitis with macrophages admixed with neutrophils and fewer plasma cells and lymphocytes. Haematoxylin and eosin staining; bar = 10 um. (**c**) Multiple *Leishmania* amastigotes within the cytoplasm of a macrophage. Arrow points to a kinetoplast at right angles to the amastigote nucleus. Haematoxylin and eosin staining; bar = 10 um.

Dog no. 1, born in the USA, presented in 2015 with ulcerative, granulomatous dermatitis affecting the nail bed of a single digit on the right forelimb which was biopsied to reveal abundant *Leishmania* amastigotes on histopathology (Figure 2) but quantitative PCR on EDTA blood at this time was negative for *Leishmania* (IDEXX Reference Laboratories, Wetherby, UK), (Table 1). Treatment over a 2-year period (2015–2017) with allopurinol (generic, China) resolved the skin disease but, in 2018, 12 months after the drug was discontinued, multiple digits on the hindlimbs developed ulcerative dermatitis. Biopsy was declined by the owner at this time, allopurinol treatment was recommenced, and is currently ongoing, and no skin disease is currently present. PCR and DNA sequencing performed at the Hebrew University in Israel on DNA extracted from paraffin-embedded tissue from the nail bed collected in 2015 and stored for five years confirmed leishmaniasis was caused by *L. infantum*. ELISA serology and PCR performed on blood collected in 2020, five years after the initial diagnosis when the dog was clinically well with no cutaneous lesions, were negative for leishmaniasis (Table 1).

**Figure 3:**
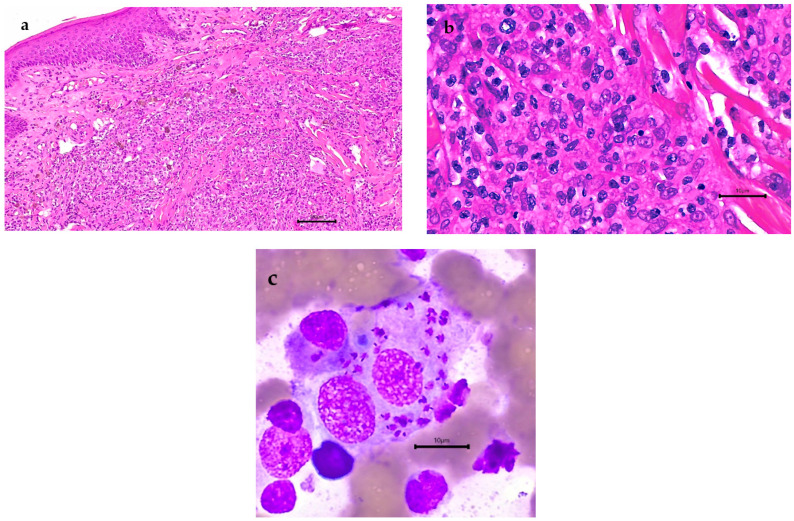
Nasal mucosa histology and splenic aspirates from dog 2: (**a**) Histopathology of nasal mucosa with granulomatous inflammation. Haematoxylin and eosin staining; bar = 100 um. (**b**) Nasal sub-mucosa with macrophages admixed with fewer neutrophils, plasma cells, and small lymphocytes. Haematoxylin and eosin staining; bar = 10 um. (**c**) Splenic aspirate. Wright’s Giemsa stain. *Leishmania* amastigotes within the cytoplasm of macrophages. Bar = 10 um.

Dog 2 was born in, and had never travelled outside of, Hong Kong (Table 1). Information about the sire and dam was unavailable, but anecdotally, the dam, born in the USA, had died at an unknown time point prior to the dog’s cutaneous disease presentation in 2018, from disease with similar manifestations to leishmaniasis. In 2018, ulcerative skin disease affecting multiple limbs as well as the nasal vestibule, causing nasal stertor, progressed to culminate in overt systemic infection in 2019 with splenomegaly, hepatomegaly, and lymphadenomegaly of the popliteal, prescapular, and sub-mandibular lymph nodes. Disease progression occurred despite various treatments involving the antibiotics cephalexin (Stada Pharmaceuticals (Asia) Limited, Kwun Tong, Hong Kong), and enrofloxacin (Dechra Veterinary Products (Australia) Pty Ltd., Somersby, NSW, Australia), the steroid prednisolone (Mavlab Pty Ltd., Slacks Creek, QLD, Australia), and the anti-fungal agent itraconazole (Europharm Lab Co., Ltd., Tai Po, Hong Kong). Incisional biopsies of the skin and nasal mucosa, collected in 2018, stained with H and E revealed pyogranulomatous inflammation within the dermis and the nasal submucosa, but no amastigotes were seen (Figure 3). GMS, PAS, ZN, Gram and Giemsa staining did not provide additional information, so clinically, leishmaniasis was not suspected. Systemic infection in 2019 manifested as lethargy and dullness with mild regenerative, normocytic, hypochromic anaemia (PCV: 31% reference range 37–54%; reticulocytes 128.1 × 10^9^/L reference range 11–92 × 10^9^/L; MCHC 321 g/L reference range 330–360 g/L), with 18 nucleated RBCs/100 white blood cells and a left shift of neutrophils (9 neutrophilic bands/100 WBCs) with a mild increase in plasma proteins (81 g/L reference range 59–78 g/L). Serum biochemistry changes included mild hyperglobulinaemia (43 g/L reference range 19–36 g/L) and hypoalbuminaemia (30 g/L reference range 32–44 g/L). There was mildly increased ALP (104 U/L reference range 17–100 U/L), AST (95.1 U/L reference range 15–57), and CK (769 U/L reference range 48–261 U/L). Spleen and liver cytology by fine needle aspirate showed granulomatous to pyogranulomatous splenitis and hepatitis with abundant *Leishmania* amastigotes within macrophages in both organs (Figure 3). Urinalysis was unremarkable.

The presence of amastigotes seen on cytological assessment of the spleen and liver prompted confirmation of leishmaniasis infection. An indirect fluorescent antibody test (IFAT) performed at the Texas A&M veterinary medical diagnostic laboratory on blood detected an antibody titre of 2048 against *L.* *infantum.* ELISA serology and PCR on blood taken two months later and performed at the Hebrew University, as well as aspirated splenic tissue, were positive for *L.* *infantum*. The ELISA result had an optical density (OD) of 1.436 (cut off 0.4 OD) and PCR, which detected the ITS-1 spacer using the ITS219 F/R primers, produced sequences from the blood and spleen which were 100% similar to one another and had 100% identity to *L. infantum* (MN503527.1) (Table 1). Dog 2 was successfully treated with allopurinol which has led to clinical resolution of all clinical signs.

The remaining six ‘in contact’ dogs (dogs 3, 4, 5, 6, 7, 8) included two dogs with chronic skin disease at the time of sampling (dogs 3 and 4) whilst the remaining four dogs (dogs 5, 6, 7, 8) were, and remain, clinically healthy. Dog 3 was born in Hong Kong and was a full sister of the systemically ill, *Leishmania*-positive dog (dog 2) (Table 1). This dog had a chronic history of alopecia with hyperpigmentation on the dorsum of the thorax and rump regions, which clinically resembled flea allergy dermatitis. Skin punch biopsies collected from alopecic regions were PCR negative for *Leishmania* and lacked amastigotes on histology on H and E, GMS, PAS, ZN, Giemsa, and Gram staining, and the histological diagnosis reported changes consistent with chronic hyperplastic dermatitis. Dog 4 was born in the Netherlands, and presented with chronic, pyogranulomatous, ulcerative dermatitis on the left stifle, but histology failed to demonstrate amastigotes or any infectious agents on H and E, GMS, PAS, ZN, Giemsa, and Gram staining, and PCR from this skin biopsy was negative for *Leishmania*. All six dogs were seronegative for *Leishmania* by ELISA and their PCR from blood and skin samples (dogs 3 and 4) was negative. 

## 4. Discussion

This report is the first to describe leishmaniasis due to *L. infantum*, in either animals or humans, in Hong Kong. The presence of leishmaniasis in the canine population is of potential public health concern as dogs are the main reservoir for human infection and canine leishmaniasis has been shown to precede human disease [6]. Canine *L. infantum* infection is often sub-clinical [7,8,9], however, clinically and sub-clinically infected dogs are infectious to sand fly vectors that can transmit infection to humans and other dogs [10,11]. Although both dogs with leishmaniasis in this report were clinically stable due to ongoing allopurinol treatment at the time of this report, it is likely that these two dogs remained infected as allopurinol, as well as all other drugs used for the treatment of canine leishmaniasis, are not known to cure dogs parasitologically and completely eliminate infection [9].

*Leishmania infantum* is transmitted by female sand fly bites [12] and only specific sand fly species serve as vectors. Although no suitable sand fly vector has been identified in Hong Kong, the natural distribution range of *Phlebotomus chinensis* Newstead, 1843, a proven vector of visceral leishmaniasis [2,13], is close to Hong Kong in the Guangdong and Hainan districts of mainland China (pers comm from senior agricultural officer from Agriculture, Fisheries and Conservation Department of Hong Kong). As geographic spread of this vector into Hong Kong is possible, the medical community needs to be vigilant about the possible emergence of leishmaniasis. 

The diagnosis of leishmaniasis in dogs 1 and 2 was unexpected. Dog 1 had been imported from the USA where leishmaniasis is not endemic but is mainly acquired during travel or military service to areas where the disease is endemic, or is transmitted predominantly via vertical autochthonous pathways [7,14,15,16,17,18]. Chronic dermatitis of the nail bed is not pathognomonic for leishmaniasis, so unless histopathology was performed, leishmaniasis was unlikely to be considered high on the differential list for this dog. 

Diagnosis of leishmaniasis in dog 2 was considered unlikely due to a number of reasons. The first is that this dog had never travelled outside of Hong Kong and leishmaniasis is not an endemic disease in Hong Kong. The second is the lack of amastigotes seen on histology of the skin and nasal mucosal lesions, which is consistent with previous reports [19,20], with one study reporting that amastigotes were only seen by histology in 20% of dogs with cutaneous disease [21]. The third reason, and the focus of this paper, is that Hong Kong lacks known vectors of this disease, so despite the fact that there was a chronically infected dog in this kennel (dog 1), autochthonous transmission was not a clinical consideration. It was not until several rounds of various treatment regimens had failed and the disease became systemic did cytology reveal abundant amastigotes in the liver and spleen. The high numbers of visceral amastigotes produced positive PCR results conducted on blood and splenic tissue and serology was also positive, confirming the diagnosis, allowing appropriate treatment to begin. 

Non-vector borne transmission of leishmaniasis in dogs has been associated with in utero infection, exposure to parasites within blood products, venereal transmission, and potentially by direct contact including dog fights [17,22,23,24,25,26,27,28,29,30,31,32] with similar incubation periods of 3 months to 7 years [26,27]. The two most likely routes of infection for dogs 1 and 2 were in utero infection and direct contact. No information was available on the sire or dam of dog 1. The dam of dog 2 and 3 died with clinical disease suspicious for leishmaniasis, but unfortunately no investigation into the cause of death was made and medical records were not available, so the date of death in relation to the diagnosis of leishmaniasis in dog 2 was unable to be determined. *In utero* infection from asymptomatic or symptomatic parents at the time of mating or whelping results in some or all puppies in a litter becoming infected [24,26,28]. It has been shown that maternal *Leishmania* disease status significantly predicts the likelihood of offspring infection in dogs [22] so it would have been interesting to know whether the dam was infected and clinically ill with *L. infantum* at the time that dog 2 and 3 were born. Dog 3, a littermate of dog 2, had no evidence of vertical transmission from the dam, being clinically healthy apart from chronic dermatitis attributable to flea allergy, and was serologically negative and PCR negative on blood and skin biopsy for leishmaniasis. 

Horizontal transmission may have occurred between dogs 1 and 2, as multiple episodes of dog fights occurred after leishmaniasis was diagnosed in dog 1. Resultant wounds bled freely and many lacerations required veterinary treatment. However, this mode of transmission has not been proven in dogs [29,30]. 

*Leishmania infantum* infection in dogs has two main patterns: sub-clinical or clinical disease, with about 5–10% of dogs naturally infected progressing to overt clinical disease [7]. The cutaneous lesion of ulcerative dermatitis affecting the nail beds of both dogs has been previously described in canine leishmaniasis [33,34]. Interestingly, although the gross lesions were similar between dogs 1 and 2, the numbers of amastigotes detected by microscopy varied, with large numbers seen on histology in dog 1, confirmed by PCR, with no obvious organisms seen in cutaneous lesions in dog 2, consistent with previous reports where the numbers of amastigotes can vary [21,33]. Large numbers of amastigotes are typically associated with exfoliative dermatitis in dogs, which was not present in either kennel dog, despite this pattern being commonly reported in canine leishmaniasis [20,33]. No amastigotes were seen in the nasal lesions from dog 2, despite amastigotes being previously reported in the nasal epithelium of infected dogs [31,35]. Dog 2 developed systemic disease with granulomatous splenitis and hepatitis as well as hyperglobulinaemia, similar to previous reports in dogs, with abundant amastigotes seen on microscopy [9,17,25,32,34,36]. Hyperglobulinaemia is associated with anti-*Leishmania* IgG antibodies, detectable via serology which are non-immunoprotective [37], and are responsible for the formation of immune-complex glomerulonephritis, which is a common cause of mortality in infected dogs [9,38]. 

This study highlights difficulties in clinically diagnosing canine leishmaniasis, especially in non-endemic geographical regions. The two kennel dogs had conflicting results on various tests during their clinical workup. Leishmaniasis in dog 1 was diagnosed on skin biopsy, but PCR remained negative on whole blood samples, collected 5 years apart, despite the abundant amastigotes seen when the first blood sample was collected. Negative PCR results from infected dogs is not surprising as, in a previous study, 25% of symptomatic dogs were negative using quantitative real-time PCR on peripheral blood [39]. Serology performed on blood 5 years after the initial leishmaniasis diagnosis was also negative, despite the dog probably remaining infected, as this dog relapsed 12 months after completing a 2-year treatment regimen with allopurinol [40,41]. Low parasite burdens due to treatment contribute to negative serological screening tests and, overall, serology fails to detect a large portion of sub-clinically infected dogs [8,42]. Skin and nasal mucosal biopsies from dog 2 failed to reveal amastigotes, but over time, amastigotes were identified on liver and splenic aspirates, confirmed by PCR on splenic aspirates and blood. Veterinarians should consider canine leishmaniasis as a potential diagnosis for skin disease in non-endemic regions and not just as a travel-acquired or imported condition. Veterinarians should also understand the limitations in the detection techniques of leishmaniasis in tissues when amastigotes are sparse in number, or on blood and serum of affected dogs when levels of circulating organisms are low. 

## 5. Conclusions

This report is the first to document autochthonous canine leishmaniasis in Hong Kong. Veterinary practitioners and public health officials need to consider leishmaniasis in local dogs from geographical areas that currently lack suitable vectors. Canine *Leishmania* infection is mostly sub-clinical, however, sub-clinically infected dogs could be infectious to sand fly vectors whose geographical range may expand, and canine infection may precede human disease [7,8,9,10,11]. Therefore, monitoring to detect phlebotomine vectors in Hong Kong is warranted and medical practitioners in non-endemic regions need to be vigilant for potential disease in humans.

## Figures and Tables

**Table 1:** Signalment, origin, main test results, and *Leishmania* treatment outcome of eight dogs living in the same kennel investigated for leishmaniasis.

Dog	Dog Country of Origin	Relationship with Other Dogs in Kennel	Year of Birth	Year of Test	Detection and Anatomic Location of *Leishmania* Amastigotes by Microscopy	ELISA Serology	PCR Result and Tissue	*Leishmania* Treatment/Outcome
1	USA	Sire of dog 5	2011	2015	Yes: Skin	ND	− (blood) *	Skin disease resolved but relapsed
				2020	ND	-	− (blood) + (skin) **	Skin disease resolved
2	Hong Kong	Full brother of dog 3	2012	2018	Suspected: Skin and nasal mucosa	ND	ND	Skin disease resolved but relapsed and became systemic
				2019	Yes: Spleen and liver	+ ***	+ (blood, spleen)	Disease resolved
3	Hong Kong	Full sister of dog 2	2012	2019	No	-	− (blood and skin)	NA
4	Netherlands	None	2009	2019	No	-	− (blood and skin)	NA
5	Hong Kong	Son of dog 1	2013	2020	ND	-	− (blood)	NA
6	Hong Kong	None	2011	2020	ND	-	− (blood)	NA
7	Sweden	None	2011	2020	ND	-	− (blood)	NA
8	Netherlands	None	2012	2020	ND	-	− (blood)	NA

− Negative result; + positive result; ND: Not done; NA: Not applicable. * Dog 1 PCR testing on EDTA blood. Quantitative *Leishmania* PCR was performed by the IDEXX Reference Laboratory, United Kingdom. All other PCRs performed by Koret School of Veterinary Medicine, the Hebrew University, Israel. ** Dog 1 PCR conducted on stored skin sample collected in 2015. *** Dog 2 had two rounds of ELISA serology; one performed by Texas A&M veterinary medical diagnostic laboratory. All other ELISA serology performed by Koret School of Veterinary Medicine, the Hebrew University, Israel.

## Data Availability

All relevant data have been included in this submission. Additional data exist, incorporated as part of confidential medical records of the attending veterinary hospital.
